# Researches on the Pathology and Treatment of Some of the Most Important Diseases of Women

**Published:** 1833-07-01

**Authors:** 


RESEARCHES ON THE PATHOLOGY AND TREATMENT OF SOME OF THE MOST IMPORTANT DISEASES OF WOMEN. By *Robert Lee*, MD. FRS. Physician Accoucheur to the British Lying-in Hospital, and the St. Mary-le-bone Infirmary. 8vo. pp. 220, Highley, London, 1833.

WE have so often expressed ourselves in terms of high commendation of Dr. Lee's professional talents, zeal, and industry, and we have so copiously reviewed in former numbers, the very admirable papers on Uterine Inflammation and on Phlegmasia Dolens, which he has contributed to the Medico-Chirurgical Transactions, that we have little to do at present, but merely to recommend this volume very strongly to the attentive perusal of those of our readers who are not in possession of the Transactions of the Society: the different papers are here collated; and some additions have been made in further illustration of those most important diseases, puerperal fever and phlegmasia dolens, or crural phlebitis, in the language of our author. The second part of the volume contains a reprint of Dr. Lee's interesting communication on the Structure of the Placenta, published in the Philosophical Transactions of last year, and noticed in our October number; and three short chapters on uterine hæmorrhage. No one has of late years deserved better of the obstetrical profession than Dr. Lee; his researches on the morbid anatomy of that disease, which under the names of puerperal fever, puerperal peritonitis, child-bed fever, epidemic disease of lying-in women, &c. has so long perplexed and bewildered medical men, have done more for the right understanding of the subject than the whole host of the violent and hot-headed publications which at different periods have issued from the press;—there is neither the antiquated bigotry of those who are determined that it is and must be a peculiar essential typhus, requiring wine, bark, and cordials for its treatment; nor yet the furious monomania of some medical reformers, whose eyes can see nothing, and whose heads can understand nothing, save inflammation of the peritoneum and viscera: the riddle is now in a great measure solved; for indeed it was staggering, especially to our young professional brethren, when they were told that equally clever men treated the same malady with brandy and ammonia, on the one hand, and with Sangrado depletion and starvation on the other. The longer we live the more do we see the strong necessity of forming ultimate conclusions in medical science, very, very slowly, and cautiously; of patient and often repeated examination of any subject before we permit our minds even to entertain any theory of its cause; and of a comprehensive analytical and synthetic review of every circumstance, whether it seems to be primary and essential, or merely secondary and collateral. If we were called upon to point out one quality of the mind more important than another, for the medical character, we should say, a freedom and simplicity of seeing things as they really are, and of looking at them with our own, instead of with other people's eyes;—a plain, common-sense, unbiassed inspection of nature, and of its phenomena; and a disregard of all flighty and rapidly-formed hypotheses. It is not bright talent, so much as a strong and vigorous tone of thinking, that is necessary for a physician;—a freedom from all prepossession, and prejudice, and a hardihood of examining for himself;—if zeal and industry be added, he cannot fail to be a useful, and perhaps a distinguished member of our profession. These remarks have been prompted by our memory of the conflicting and utterly discordant statements of different writers on the subject of puerperal fever. Thanks to Dr. Lee, he has in a great measure furnished us with the clue to conduct us through the labyrinth of opinions, and has taught us how to reconcile, at least to a certain extent, assertions apparently distinct, as far as East is from the West. He most satisfactorily proves, that all the destructive febrile affections which follow parturition, are invariably associated with, if not directly caused by, inflammation of some of the textures of the womb, or of its appendages; and that the type or character of the fever is probably dependent upon the particular tissue most involved; thus, that in the inflammatory pyrexia, the peritoneal lining chiefly is inflamed; in the congestive, the muscular substance, and in the low typhoid, the veins of the uterus and ovaria. This is the pith and marrow of Dr. Lee's doctrines; the corner-stone of his ingenious, and we may probably add, secure building. Our readers will do well to look back to our number for Oct. 1831, where they will find the details at full length.*

* Into the literary controversy between Drs. Granville and Lee, we do not mean to enter. They are "Arcades ambo—et respondere parati." This article was written long before the contest began; and, as they are both our personal friends, we must decline the disagreeable office of umpire. The public is the supreme judge, and journalists are, at the best, but special pleaders.—Ed.

The section which treats of uterine phlebitis merits a most careful and studious perusal. The all-comprehensive mind of John Hunter was the first to point out that veins are subject to inflammation and its consequences, and that, very often, most dangerous and fatal symptoms result from these morbid states. Three years after his paper was published, the Italian surgeon Paletta made some very interesting observations in his "Exercitationes Pathologicæ, 1787."

"Grave adeo ac vehemens malum non videbatur in sanguineis pelvis vasis substitisse: sed humorem per venæ cavæ torrentem ad cor delatum, in remotiori aliqua parte depositum fuisse, jure suspicabamur. Quære reserato thorace in dentro pulmone, qui undique liber, colore et consistentia naturali erat, quatuor abscessus offendimus," &c.

. . . "Hæc enim (vasa) sive saniosam materiam ex ipso ulcere exceptam ad interiores partes deportarint, sive quod verosimilius, pus ob tunicarum inflammationem in earum lumine generatum a redeunte sanguine in humorum massam transvectum sit, certe utrovis modo ab extremis partibus ad interiores per hac vasa materia peccans delata est.

. . . . "Si itaque hac transvectio causa est apostematum in memoratis visceribus observatorum: nonne idem sentiendum est de abscessibus, qui post graves capitis lesiones in hepate, liene, pulmone, pericardio consequuntur? Possunt utique sanguineæ venæ ob ictus vehementiam et capitis concussionem, etc. inflammationi ut aliæ partes esse obnoxiæ," &c. 58.

In addition to the researches of Meckel, Dr. John Clarke, and Mr. Wilson, Professor Burns of Glasgow, in his Principles of Midwifery, observes, in the chapter of his work on Inflammation of the Uterus,—

"That pus is often contained in the ovaria and tubes, and sinuses of the uterus. Mortification is an extremely rare termination. This is a fact of which my dissections convince me, and it is further confirmed by the opinion of Dr. Clarke. Little or no serous effusion takes place into the abdomen. In some cases the veins participate very extensively in the disease, and become inflamed to a great distance. Thus, inflammation may spread toward the heart or liver, or down along the veins of one or both thighs. This is attended with great and debilitating fever, and much pain in the course of the affected veins, which after death are found inflamed, thickened, or filled with pus."*

It is to be remembered, however, that Dr. Burns describes puerperal peritonitis and uteritis as diseases essentially distinct from puerperal fever, in which, he says, "that the womb, although sometimes the first seat of the pain, and occasionally found inflamed, is in general not more affected than the intestines." In 1826, M. Louis reported a fatal case of malignant puerperal fever, in which he found, upon dissection, the uterine veins diseased; and M. Dance the same year published the histories of several cases in his Inaugural Dissertation at Paris. It was not however till 1829, that Dr. Lee became acquainted with the labours of his predecessors, and his first memoir on the subject had been previously read to the Medical and Chirurgical Society of London; so that he is fully entitled to share the merit of the discovery, that uterine phlebitis is often associated with many of the worst cases of puerperal fever. It is right here to state, that much praise is due to Mr. Arnott also, for his very valuable paper "on the secondary effects of the inflammation of the veins," which our readers will find copiously reviewed in

* The Principles of Midwifery, by J. Burns. London, 1820, p. 524.

our 23rd No., and to which we, although differing from the author in some very important particulars, awarded high commendation. Dr. Lee himself candidly avows "that, before hearing the observations of Mr. Arnott, I was entirely unacquainted with the true cause of several of the most severe constitutional symptoms of uterine phlebitis." We cannot afford space for further remark, either on this topic or on the sections which treat of the causes and treatment of the disease; one paragraph, however, is too important to be omitted.

"From what has fallen under my own observation in the British Lying-in Hospital, and other similar institutions in this metropolis, where the utmost attention is paid to ventilation and cleanliness, and where the wards are not overcrowded with patients, I cannot hesitate to express my decided conviction, that by no means hitherto discovered, can the frequent and fatal recurrence of the disease be prevented in Lying-in Hospitals, and that the loss of human life thereby occasioned, completely defeats the objects of their benevolent founders." 115.

The next division of Dr. Lee's work is occupied with the investigation of phlegmasia dolens, as it used to be called in the good old times; for soon we suppose the term will be obsolete, and not to be understood, so sweeping threatens to be the besom of reform. Our author prefers the appellation of crural phlebitis to that of "phlegmasia dolens, œdema lacteum, depots laiteux, and the other hypothetical names which have, up to the present time, been employed to designate this disease; because the swelling of the affected limb, and all the other local and constitutional symptoms of the affection, invariably depend on inflammation of the iliac and femoral veins." We do not think that there is wisdom in this change: what hypothesis, in sooth, is associated with the term "phlegmasia dolens," or painful inflammatory swelling? it is a simple predication of certain phenomena which are present in every case; nothing, it seems to us, could be more appropriate and befitting. Our readers are no doubt prepared to expect that we should object to the name of crural phlebitis, and for this very good reason, that we are not yet satisfied that it has been clearly made out that the disease consists in inflammation of the veins alone. We cannot enter at large into a detail of the data and reasoning on which our scepticism is founded, but refer to many of our preceding Numbers, as No. 2, where a review of Dr. David Davis' original paper will be found, and Nos. 18, 22, and 23. The single fact, admitted by Dr. Lee himself, that the veins of the groin and extremity are sometimes violently inflamed, and yet no symptoms of phlegmasia have been present during life, is sufficient to make us pause a little longer, before we christen the disease with a new hypothetical name. Besides, what reasonable man objects to such appellatives as typhus, apoplexia, epilepsia, hysteria, and so forth, because they do not specify the morbid states which, it is supposed, excite the diseases in question?

We shall very briefly enumerate our reasons for not agreeing with the doctrine of the phlebitic origin of true phlegmasia dolens; they are as follows:—Because the inflammation of the veins may be present, without any of the symptoms of the disease; *vide*, among others, a paper by Mr. Wilson, in the Transactions of a Society for the Improvement of Medical and Chirurgical Knowledge, vol. II.;—because genuine phlegmasia dolens is rarely fatal, and genuine phlebitis is one of the most destructive of all diseases—

Dr. W. Hunter, speaking of the former, says "it generally does well;" in 14 cases recorded by Mr. White, of Manchester, not a patient died; in six mentioned by Mr. Frye of Gloucester, in his Essay, 1792, recovery took place; whereas, in 15 cases enumerated by Dr. Lee, eight proved fatal;—3rdly, because we are of opinion that many cases are admitted into the catalogue of phlegmasia dolens, which do not strictly belong to this disease; else how are we to account for the much increased frequency in modern reports? formerly it was a rare disease, but there is no lack of examples of this crural phlebitis now-a-days;—fourthly, because we do not meet with that train of gloomy constitutional symptoms, nor the purulent deposits in the lungs, liver, joints, cellular membrane, &c., which so very generally attend ordinary phlebitis. It is also to be remembered, that pus has seldom been found in any of the inflamed veins of patients who have died of phlegmasia dolens; how different is this from the morbid phenomena of the veins of the arm, when phlebitis supervenes to venesection, or of the spermatic and uterine veins in many cases of puerperal fever, recorded by Dr. Lee in this very volume. Surely, therefore, we cannot regard the pathological state of all these diseases as identically the same;—we do not deny that, in every case of plegmasia, the hypogastric, iliac, and crural veins may be inflamed; for aught that we know to the contrary, this may be true; but we believe that other tissues, as well as the venous, are affected, and that, unless they were, we should not have the phenomena of genuine phlegmasia dolens. Besides the preceding arguments, we may allude to the discoloration of the skin, to the œdema of the cellular texture, to the chain of little abscesses, which are so very commonly attendants on genuine phlebitis, and which do not belong to the old orthodox phlegmasia dolens. We are, therefore still inclined to adopt the opinion of Dr. Hall, that the disease consists of a peculiar inflammation, seated in the muscles, cellular membrane, and inferior surface of the skin, producing a rapid effusion of coagulable lymph; and that the obstructions, or other organic changes, found in the veins or lymphatics are to be considered as secondary effects, and not as a primary causes: and, at the same time, to adhere to what we have put on record in February, 1830—that "Dr. Lee and others have proved that many cases, so closely resembling phlegmasia dolens as hardly to admit of being distinguished from it, are essentially inflammation of the iliac and femoral veins; and they have rendered it probable, that the majority of the instances of that affection are dependent immediately, or remotely, on venous inflammation."

Before leaving this subject, it is proper to state that Dr. Lee is of opinion, that the phlebitis, in phlegmasia, invariably commences in the uterine veins, and is thence propagated along the branches of the hypogastric to the internal, common, and external iliacs, to the femoral vein and its branches. The cases adduced by him render this supposition very probable, but do not quite warrant us in laying it down as of universal occurrence, and we require further data before we can say more. We are to recollect that, in some instances, the pain and swelling begin in the calf of the leg, and mount upwards to the goin. Dr. David Davis is still of opinion that the inflammation commences in the common iliac, and not in the uterine veins, and that the disease is produced by the pressure of the gravid uterus. It does not appear, however, that any examination of the hypogastric veins was made in

Dr. D.'s cases; and the circumstance of crural phlebitis being an occasional consequence of suppressed catamenia and of structural changes of the uterus, adds probability to the correctness of the view taken by Dr. Lee. Four cases are reported in illustration of the former cause, and the same number to shew the effects of cancerous ulceration of the cervix uteri on the uterine and other adjacent veins; a very interesting example of this affection is related by Mr. Lawrence, in the 16th volume of the Medical and Chirurgical Transactions, p. 59. The following appearances were found on dissection.

"The hypogastric vein, involved in the diseased mass, was closed, in consequence of previous inflammation of its coats; and the same change had occurred in the internal iliac, the common iliac, the external iliac, the femoral and profunda veins, as well as in the internal saphena; all of which were completely impervious. The affection terminated above at the junction of the common iliac vein with that of the opposite side; the latter vessel and the inferior cava being quite natural. The saphena was closed for a length of about four or five inches beyond which it was natural. The profunda was cut through near the femoral vein, and the latter was divided as it passes the tendon of the triceps. The disease extended in both these vessels beyond the situations where they had been divided; but its inferior limits were not ascertained. The right spermatic vein was closed in its lower half." 162.

*Crural phlebitis, in man,* may be induced by disease of the rectum, bladder, prostate, urethra, &c. the inflammation commencing in the veins of these parts, and extending gradually to the iliac and femoral vessels. Mr. Lawrence mentions a case of phlebitis, where it occurred in a patient who laboured under cancer of the rectum; Mr. Holberton reports two—the patients died of diarrhœa and ulcerations of the bowels. Dr. Cheyne "observes, in his report of the Whitworth Hospital, which contains an account of dysentery, that 'it is worthy of remark, that a swelling occurred in several of the patients, both males and females, resembling the phlegmasia dolens in all respects but in its connexion with parturition.'" Drs. Tweedie, Graves, and Stokes relate cases of painful swellings of the lower extremities, closely resembling phlegmasia dolens, after fevers; and M. Cruveilhier mentions that of a man, in whom the symptoms came on after the introduction of a sound into the bladder for retention of urine, occasioned by an enlarged prostate.

One of the most common causes is the application of cold to the limb. In the case of the late Earl of Liverpool, recorded by Sir H. Halford, the attack was induced by exposure to a current of cold air, which passed through an open window upon the lower extremities, when but thinly clothed, while his Lordship was attending a crowded levee. We shall transcribe the following very interesting case, communicated by Drs. Graves and Stokes.

"A young man of a strong habit was employed for two successive days in working a ditch, and was consequently obliged to stand in water above his knees during that time. On the following day he became affected with lassitude, vertigo, and general weakness, and complained of severe pain in the right thigh. These symptoms continued for seven days, when he was admitted into the Meath Hospital. His countenance was anxious and depressed. The tongue furred, thirst, headache, urine scanty, turbid, and high-coloured. Pulse 96. Skin mottled with petechiæ. In addition to these general symptoms, the respiration was laboured and unequal, with some cough; face very livid. But his chief complaint was a severe pain in the upper and anterior part of the right thigh, which was greatly aggravated by motion or pressure. He had also severe pain in the left hypochondrium.

At this time no swelling of the limb whatever could be detected, but in the course of two days the upper portion of the thigh became evidently swollen, the part being extremely tender, but not at all red. The pain of the side continued, and extensive bronchial and pneumonic inflammation was detected. General bleeding and very free leeching to the limb was employed. The blood was not inflammatory, and no relief was experienced by the patient. The swelling of the thigh increased, calomel and opium were freely exhibited, but without any effect. The typhoid symptoms increased, and the patient died on the fourth day after his admission.

On dissection the right lower extremity was found to be tense and swollen in its superior portion, while the leg and foot were slightly anasarcous. The sac of the pericardium contained some sero-purulent fluid, and that portion covering the auricles and great vessels was vascular, and in many places covered with coagulable lymph. Both lungs were in a state of extreme sanguineous congestion, with commencing solidity in their posterior inferior portion, and general inflammation of the pleura. The bronchial mucous membrane was universally red, and the tubes filled with frothy mucus.

The vena cava contained a few portions of a substance of a granular appearance, friable, and of a yellowish colour. This did not adhere to the vessel, which otherwise appeared healthy. In the external iliac vein, however, just above Poupart's ligament, a large concretion of a similar nature, nearly plugging up the vessel, and extending into some of the minute collateral branches. The lining membrane red, and in one point adhered to the coagulum. No puriform matter could be detected. The femoral and popliteal arteries were healthy. The cellular tissue of the limb was œdematous." 168.

Similar swellings are also occasionally brought on by the irritation of varicose ulcers on the limbs; and their occurrence is by no means unfrequent in the upper extremities of women afflicted with cancerous mammæ. Laennec has made the important observation.

"It is not uncommon to find the veins in the neighbourhood of a cancerous breast filled with pus, either pure or mixed with blood, sometimes fluid, at other times of the degree of consistence of an atheromatous tumour.

An additional consequence of the presence of too much pus in the blood, is the production of inflammation in different organs, and especially the lungs, which runs rapidly into suppuration. It is from this circumstance, that the subjects of surgical operations, and those labouring under extensive suppurations, are frequently cut off by peripneumonies, which, according to the observations of M. Cruveilhier, are usually lobular, that is, commencing in several points at once. This, in my opinion, is the mode in which we must explain the occurrence of metastasis of pus, at least, in the majority of cases."* 72.

With this we close our review of the first part of Dr. Lee's researches. Part II. professes to treat of uterine hæmorrhage; but the greater portion of it is occupied with a reprint of his very valuable paper on the structure of the placenta. The facts there stated appear to warrant the conclusions —that the placenta does not consist (as all anatomists have hitherto described) of two parts, the maternal and the fœtal;—that no cells exist in its substance;—that there is no communication between the uterus and the

---

* Laennec on Diseases of the Chest, by Forbes, 1827, p. 652.

placenta by large arteries and veins;—and that therefore whatever changes take place in the fœtal blood must result from the indirect exposure of this fluid, as it circulates through the placenta, to the maternal blood flowing in the great uterine sinuses; seeing that the deciduous membrane is interposed between the one set of vessels and the other. Professor Burns, of Glasgow, has published a paper, last July, in the Medical Gazette, in which he advocates the old doctrine of a direct communication of the large uterine and placental vessels, and of the cellular texture of the placenta; but we do not think that he has been successful in refuting the interesting discoveries of Dr. Lee. As this is a subject of great interest to the anatomist and physiologist we are tempted to find room for the valuable communications of Mr. Owen, of the College of Surgeons, whose authority must weigh highly in all anatomical researches, and of Mr. Charles Millard, the Demonstrator of Anatomy in the School of Webb Street.

"My Dear Sir—During the time you were examining the Hunterian preparation of the uterus and placenta in the Museum of the Royal College of Surgeons, your observations on the obscurity produced by the extravasated injection led me to think of some less objectionable mode of demonstrating the vascular communication between the uterus and placenta, if it existed; or of proving more satisfactorily than the appearances you pointed out in that preparation seemed to do, that there was no such communication.

You have since afforded me the means, through the kindness of Mr. Alex. Shaw, of examining, in the manner I wished, the anatomical relations between the placenta and uterus. This has been done by dissecting the parts under water before disturbing them, either by forcibly throwing foreign matter into the vessels, or by separating the placenta from the uterus, to observe the appearances presented by the opposed surfaces,—a proceeding which, if done in the air is liable to the objection of the possibility of having torn the vessels which were passing across, and the coats of which are acknowledged by those who maintain the existence of such vessels, to be extremely delicate.

The mode, therefore, which was adopted to avoid these objections, was to fix under water, in an apparatus used for dissecting mollusca, &c. a section of the uterus and placenta, and, commencing the dissection from the outside, to remove successively and with care the layers of fibres, and trace the veins as they pass deeper and deeper in the substance of the uterus in their course to the deciduous membrane; in which situation, as the thinnest pellicle of membrane is rendered distinct by being supported in the ambient fluid, I naturally hoped in this way to see the coats of the veins continued into the deciduous membrane and placenta, and to be able to preserve the appearance in a preparation, if it actually existed in nature. But in every instance, the vein, having reached the inner surface of the uterus, terminated in an open mouth on that aspect; the peripheral portion of the coat of the vein, or that next the uterus, ending in a well-defined and smooth semicircular margin, the central part adhering to, and being apparently continuous with, the decidua.

In the course of this dissection, I observed that where the veins of different planes communicated with each other, the central portion of the parietes of the superficial vein invariably projected in a semilunar form into the deeper-seated one; and where (as was frequently the case, and especially at the point of termination on the inner surface) two or even three veins communicated with a deeper-seated one at the same point, these semilunar edges decussated each other, so as to allow only a very small part of the deep-seated vein to be seen. I need not observe to you how admirably this structure is adapted to ensure the effect of arresting the current of blood through these passages, upon the contraction of the fibres with which they are every where surrounded.

On another portion of the same uterus and placenta, (which were removed from a woman who died at about the fifth month of utero-gestation,) I commenced the examination under water by turning the placenta and deciduous membrane from the inner surface of the uterus. In this way, the small tortuous arteries that enter the deciduous membrane were readily distinguishable, though not filled with injected matter; and as it was an object to avoid unnecessary force in the process of separation, they were cut through, though they are easily torn from the decidua. But with respect to the veins, they invariably presented the same appearances as were noticed in the first dissection, terminating in open semi-circular orifices, which are closed by the apposition of the deciduous membrane and placenta. This membrane is, however, certainly thinner opposite these orifices than elsewhere; and in some places appeared to be wanting, or, adhering to the vein, was torn up with it; but in these cases the minute vessels of the placenta only appeared, and never any indication of a vascular trunk or cell commensurate with the size of the vein whose terminal aperture had been lifted up from the part.

The preparation which accompanies this letter shows the termination of a vein on the inner surface of the uterus, and an artery of the decidua cut through, with the corresponding appearances on the surface of the placenta; also the valvular mode in which the veins communicate together in the substance of the uterus.

I remain yours, very truly, RICHARD OWEN." 197.

Mr. Millard states—

" The parts were examined without any previous injection or other preparation, that every thing might be seen in its natural state. On making an incision through the anterior wall of the uterus, the attention was immediately arrested by the large size of the uterine veins, especially of those in the neighbourhood of the placenta. The right side of the anterior wall of the uterus was then carefully turned back, and with such ease as to convince me that no large vessels were torn through; the tunica decidua was now distinctly seen passing behind the placenta, and it was also observed to pass over the orifice of the fallopian tube. The other side of the uterus was then carefully examined under water, principally with a view to determine the direction and termination of the uterine veins, and the connexion that exists between the uterus and placenta. This examination completely coincides with your description. The uterine veins passed in an oblique direction, as regards the placenta, and not immediately towards it, and in no instance could they be traced into its structure, for whether they were followed from the external to the internal surface of the uterus, or in the opposite direction, they were found to present a number of large valvular openings, some of an elliptical and some of a semicircular form, situated in the sides of the veins, and having no corresponding openings on the outer surface of the placenta, but closed by the deciduous membrane. All these openings had distinct, well-defined edges, formed, apparently, by a duplicature of the lining membrane of the uterus, and quite unlike ruptured vessels; indeed, as I have before stated, none of these veins could be followed into the placenta, even by the most careful examination. But both arteries and veins, not larger than a bristle, were readily traced from the surface of the uterus to the tunica decidua covering the uterine surface of the placenta, where they ramified very minutely. Some of these were distended by inflating the large uterine veins, but no air could be made to pass from these vessels into the substance of the placenta, although the inner membrane was distinctly raised by it. The uterus was farther connected to the placenta by a quantity of pulpy cellular membrane, which easily broke down under the finger." 202.

In uterine hæmorrhage, when the placenta is situated over the os uteri, Dr. L. makes the important remark, that the flooding is always increased by the labour-pains, and generally also during the examination with the finger; whereas the reverse of this takes place in other hæmorrhages, which are invariably more or less abated during each contraction of the uterus. In one case the placenta was expelled first, the child following;—the woman nearly perished. Several other cases are adduced in our review of Dr. Ramsbotham's Midwifery. When the flooding comes on after the separation and removal of the placenta, our chief reliance, in the opinion of the author, ought to be in powerful pressure on the hypogastrium, and in the external application of napkins plunged into cold water, and suddenly dashed on the abdomen; ice introduced into the vagina and applied in a bladder to the pubis, is also recommended. The practice of introducing the hand into the uterus, to stimulate it to contraction, "is not only often ineffectual for this purpose in the worst cases of flooding, but that it often gives rise to subsequent fatal inflammation of the deep-seated structures of the uterus. I have repeatedly passed the hand into the uterus to produce contraction, but it has refused to obey the stimulus of the hand; it has remained like a soft flaccid bag—more like a piece of intestine than uterus, and the blood has continued to pour down the arm, until the hand has been withdrawn and more efficient remedies employed." 216.

The testimony of Dr. Dewees is brought forward in confirmation; he says, "that he can with most perfect truth declare that he has not found it necessary to introduce the hand for the purpose of stopping an hæmorrhage after the expulsion of the placenta, for more than the last five-and-thirty years, and that he regards the practice as always frightful, and oftentimes unnecessary and pernicious." 216.

Our experience is however strongly in favour of the practice, and the high authority of Professor Burns may be adduced. "The hand is to be immediately introduced into the womb, and must be kept there, moving it gently, until the fibres contract; and until this takes place, neither the hand nor the placenta should be withdrawn," p. 396; and again, speaking of hæmorrhage after the expulsion of the placenta—"the only security consists in uterine contraction; this is to be excited by the application of cold, and by the introduction of the hand; not simply to extract the coagula, but to stimulate the uterus, and rather make it expel them." P. 401. With regard to the ergot of rye in such cases, Dr. Lee is quite sceptical of its good effects, whether the placenta has been expelled or not: and his stricture on the proposal of Plouquet, to interrupt the circulation through the abdominal aorta, is severe, but just.

"Whoever it was who first recommended the introduction of the hand into the uterus to compress the aorta, he must have been alike ignorant of the structure of the gravid uterus, and of the process employed by nature to suppress uterine hemorrhage. The hand, if applied to compress the aorta through the uterus, would be placed over it, below the origin of the spermatic arteries, which supply that part of the uterus where the placenta usually adheres. Pressure over the lower part of the aorta might prevent the flow of blood into the iliac arteries, but it could not fail to increase the hemorrhage, by forcing the blood still more strongly into the vessels from which it was flowing in the upper part of the uterus." 217.

In taking leave of Dr. Lee's work, we feel it to be alike our pleasure and duty once more to record our opinion of its high and sterling merits;—it ought to have a place on the shelves of every physician in the kingdom.

